# An Angiotensin II Type 1 Receptor Blocker Prevents Renal Injury via Inhibition of the Notch Pathway in Ins2 Akita Diabetic Mice

**DOI:** 10.1155/2012/159874

**Published:** 2012-01-29

**Authors:** Masaya Koshizaka, Minoru Takemoto, Seiya Sato, Hirotake Tokuyama, Masaki Fujimoto, Emiko Okabe, Ryoichi Ishibashi, Takahiro Ishikawa, Yuya Tsurutani, Shunichiro Onishi, Morito Mezawa, Peng He, Satoshi Honjo, Shiro Ueda, Yasushi Saito, Koutaro Yokote

**Affiliations:** ^1^Department of Clinical Cell Biology and Medicine, Chiba University Graduate, 1-8-1 Inohana, Chuo-ku, Chiba-shi, Chiba 260-8670, Japan; ^2^Division of Diabetes, Metabolism and Endocrinology, Department of Medicine, Chiba University Hospital, 1-8-1 Inohana, Chuo-ku, Chiba-shi, Chiba 260-8670, Japan; ^3^Seiwa Narashino, 3-5-3 Akitsu, Narashino-shi, Chiba 275-0025, Japan; ^4^Ueda Clinic, 1-13-18 Nobuto, Chuo-ku, Chiba-shi, Chiba 260-0032, Japan; ^5^Chiba University, 1-33 Yayoi-cho, Inage-ku, Chiba-shi, Chiba 268-8522, Japan

## Abstract

Recently, it has been reported that the Notch pathway is involved in the pathogenesis of diabetic nephropathy. In this study, we investigated the activation of the Notch pathway in Ins2 Akita diabetic mouse (Akita mouse) and the effects of telmisartan, an angiotensin II type1 receptor blocker, on the Notch pathway. The intracellular domain of Notch1 (ICN1) is proteolytically cleaved from the cell plasma membrane in the course of Notch activation. The expression of ICN1 and its ligand, Jagged1, were increased in the glomeruli of Akita mice, especially in the podocytes. Administration of telmisartan significantly ameliorated the expression of ICN1 and Jagged1. Telmisartan inhibited the angiotensin II-induced increased expression of transforming growth factor **β** and vascular endothelial growth factor A which could directly activate the Notch signaling pathway in cultured podocytes. Our results indicate that the telmisartan prevents diabetic nephropathy through the inhibition of the Notch pathway.

## 1. Introduction

The worldwide prevalence of diabetes in all age groups was 2.8% in 2000 and is estimated to be 4.4% in 2030 [1]. The total number of people with diabetes mellitus (DM) is expected to rise from 171 million in 2000 to 366 million in 2030. Diabetic nephropathy, a major microvascular complication of DM, is the most common cause of end-stage renal disease (ESRD) [2]. The number of ESRD cases is expected to increase mainly as a result of the increasing incidence of obesity and type 2 DM.

A number of pathways such as the protein kinase C pathway [3] and the polyol pathway [4] as well as advanced glycation end products [5] have been reported to play important roles in the development of diabetic nephropathy. It has also been reported that the renin-angiotensin system (RAS) plays a potent role in the initiation and progression of diabetic nephropathy [6].

A number of clinical evidences have suggested that the blockade of the RAS by angiotensin-converting enzyme (ACE) inhibitors (ACEIs) and/or angiotensin II type1 receptor (AT1R) antagonists (ARBs) could improve renal function or slow down disease progression in diabetic nephropathy [7]. Furthermore, it has been reported that ACEIs and/or ARBs inhibit the RAS and have pleiotropic effects, which improve renal prognosis.

Recently, Niranjan et al. reported that the Notch pathway was activated in diabetic nephropathy and in focal segmental glomerulosclerosis (FSGS) [8]. The activation of the Notch pathway in podocytes has been studied in genetically engineered mice. These mice developed glomerulosclerosis due to the activation of p53, which induced apoptosis in podocytes. The same group also showed that pharmaceutical and genetic blockade of the Notch pathway prevented mice from developing diabetic and puromycin-aminonucleoside- (PAN-) induced glomerulosclerosis.

The Notch signaling pathway is a signaling pathway that determines cell fate [9]. Further, it is regulated by cell-cell communication during the formation of various internal components such as the nerves, blood, blood vessels, heart, and hormonal glands. Notch is a transmembrane receptor protein that interacts with ligands of the Jagged and Delta families [10].

The aim of this study was to examine the activation of the Notch pathway in Akita mice as well as the effects of telmisartan on the Notch pathway both *in vivo* and *in vitro*. 

## 2. Materials and Methods

### 2.1. Reagents

Telmisartan was obtained from Nippon Boehringer Ingelheim Co., Ltd. (Tokyo, Japan). Candesartan was purchased from Tronto Research Chemicals (North York, Canada). Angiotensin II was obtained from Sigma-Aldrich (St. Louis, MO). Recombinant human TGF-*β*1 (#240-B) and recombinant human VEGF-A (#293-VE) were purchased from R&D systems (Minneapolis, MN). GSI was purchased from Calbiochem (San Diego, CA). Hoechst 33342 was from Dojindo laboratories (Kumamoto, Japan).

### 2.2. Animals

Male heterozygous Ins2 Akita diabetic mice (C57BL/6) and C57BL/6 controls were obtained from Japan SLC Inc. (Shizuoka, Japan). Eight-week-old Akita mice and control mice received telmisartan (5 mg·kg^−1^·day^−1^) or no treatment for 15 weeks (*n* = 8 in each group). The blood glucose level, body weight, blood pressure, and urinary albumin excretion were measured every two weeks. The blood glucose level was examined using Medisafe-Mini (TERUMO Corporation, Tokyo, Japan), and the blood pressure was determined by the tail cuff method using Softron BP-98A (Softron, Tokyo, Japan). In order to estimate albuminuria, mice were individually housed in metabolic cages for 24 h. Urine was collected, and urinary albumin concentrations were measured with a Lebis Albumin assay kit (Shibayagi, Gunma, Japan). The blood creatinine levels, BUN, fasting blood glucose levels, and HbA1c were measured at the time of sacrifice. All experiments in this study were performed in accordance with the Guidelines of the Animal Care and Use Committee of Chiba University, Japan, which follows the Guide for the Care and Use of Laboratory Animals (NIH publication no. 85-23, revised 1985). The ethics committee for animal research at Chiba University approved all animal experiments.

### 2.3. Immunohistochemistry

The following commercially available antibodies were used: rabbit anti-Jagged1 (1 : 200 dilution, sc-11376) and rabbit antihuman TGF-*β*1 (1 : 50, sc-146) antibodies were purchased from Santa Cruz Biotechnology (Santa Cruz, CA). Rabbit anti-cleaved Notch1 antibody (1 : 100, Val1744, no. 2421S) was purchased from Cell Signaling (Danvers, MA). Rat anti-podocalyxin monoclonal antibody (0.5 *μ*g/mL, MAB1556) was from R&D systems. Mice kidneys were embedded in OCT compound and frozen, and 10 *μ*m sections were made. The sections were air dried, fixed in methanol (10 min on ice), rinsed in phosphate-buffered Tween (PBT), and blocked for 30 min with phosphate-buffered saline (PBS) containing 0.5% bovine serum albumin (BSA). Primary antibodies were diluted in PBS containing 1% BSA and were incubated with the sections overnight at 4°C. The slides were rinsed with PBT for several times. The fluorophore-conjugated secondary antibodies were applied for 2 h. The sections were again rinsed with PBT for several times, mounted (Vectashield Mounting Medium with DAPI; Vector Laboratories, Inc., Burlingame, CA), and viewed under a fluorescence microscope (Axio Observer; Leica) or a confocal laser scanning microscope (Leica LSM5 PASCAL). The images were processed using Adobe Photoshop.

### 2.4. Cell Culture

Mouse podocytes, conditionally immortalized with a temperature-sensitive variant of the SV40 large T-antigen, were kindly provided by Dr. Peter Mundel (Albert Einstein College of Medicine, NY, USA). The preparation and characterization of these cells have been described elsewhere [11]. Podocytes were maintained in Roswell Park Memorial Institute (RPMI) 1640 medium (Gibco/Life Technologies, Grand Islands, NY, USA) supplemented with 10% fetal bovine serum (FBS; Sigma Aldrich), 100 U/mL penicillin, and 100 U/mL streptomycin (Sigma Aldrich). To propagate podocytes, cells were cultivated at 33°C and incubated with 10 U/mL of murine recombinant *γ*-interferon (Pepro Tech EC Ltd, London, UK) to enhance the expression of the T-antigen (permissive conditions). To induce differentiation, podocytes were cultured at 37°C without *γ*-interferon in RPMI 1640. Cells were cultured under nonpermissive conditions for at least 11 d before they were used in the experiments. The medium was changed every 3 d to induce full differentiation. Cells at passages 12 to 18 were used for the experiments in this study.

### 2.5. Reverse Transcriptase-Polymerase Chain Reaction

The expression of mRNA in podocytes was analyzed by reverse transcriptase-polymerase chain reaction (RT-PCR). Total RNA was extracted using an RNeasy Mini Kit (Qiagen, Hilden, Germany) according to the manufacturer's instructions. After treatment with DNase, 1 *μ*g of total RNA was reversely transcribed using oligo dT primer, pd(T)12–18 (Invitrogen, Carlsbad, CA), to avoid genomic contamination. The cDNA was generated using SuperScript III Reverse Transcriptase (Invitrogen, Carlsbad, CA). Gene-specific oligonucleotides for the PCR analyses were designed according to the predicted cDNA sequences (http://www.ensembl.org/). The PCR was performed in a 25 *μ*L PCR reaction containing 1 *μ*L of complementary DNA (cDNA), Taq reaction buffer (Go Taq, Promega, Madison, WI), and 10 *μ*M of dNTPs. The primer sequences and sizes of the expected PCR products are as follows: Hes1, 5′-CCCTGTCTACCTCTCTCCTT-3′, 5′-AGGTGCTTCACAGTCATTTC-3′, 472 bp; TGF-*β*, 5′-TCCAAGAAAAAGAAAATGGA-3′, 5′-CTCTGAATCAGGTTGTGGAT-3′, 452 bp; VEGF-A, 5′-GTGGACATCTTCCAGGAGTA-3′, 5′-ATCTGCAAGTACGTTCGTTT-3′, 382 bp; *β*-actin, 5′-TCGTGCGTGACACATCAACATCAAAGAG-3′, 5′-TGGACAGTGAGGCCAGGATG-3′, 411 bp. PCR was performed for 25–30 cycles. Each cycle consisted of denaturation at 94°C for 2 min, annealing at 50°C for 30 s, and extension at 72°C for 30 s. PCR amplification was followed by a final extension step at 72°C for 7 min. An aliquot of 10 *μ*L of each PCR product was subjected to electrophoresis on a 2% agarose gel (Ronza), followed by staining with an ethidium bromide solution (Sigma). The signals were photographed with a charge-coupled device (CCD) camera system (Printograph, ATTO). Densitometric analyses of the fluorograms were performed using an image scanner (EPSON GT-X900) with ImageJ software (http://rsbweb.nih.gov/ij/download.html).

### 2.6. Morphometric Analysis

Five glomeruli (*n* = 3, in each) were randomly selected from each specimen. The extent of extracellular mesangial matrix was determined by quantification of the periodic-acid-Schiff-staining- (PAS-) positive area in the mesangium and divided by the glomerular tuft area. The extracellular mesangial matrix area and glomerular tuft area were quantified by ImageJ.

### 2.7. Detection of Apoptosis by Hoechst Staining and Flow Cytometric Assays

Podocytes were treated with AII in the presence or absence of telmisartan for 72 h. After the treatment, apoptosis was defined as the presence of nuclear condensation on Hoechst staining. Alternatively, the cells were collected, washed twice with cold phosphate-buffered saline (PBS), and centrifuged at 1,000 g for 5 minutes. Subsequently, the Annexin V/propidium iodide assay was carried out to determine apoptosis according to the manufacturer's instructions (BD Pharmingen) and analyzed by flow cytometry (FACSCalibur; BD Immunocytometry Systems, San Jose, CA).

### 2.8. Statistical Analysis

Results are expressed as the mean ± standard error of the mean (SEM). Experimental points were performed in triplicates with a minimum of three independent experiments. An unpaired Student's *t*-test was used for comparison of two groups. *P* < 0.05 was considered significant. 

## 3. Results

### 3.1. Telmisartan Reduces the Urinary Albumin Excretion in Akita Mice

First, we evaluated the effect of telmisartan on blood pressure in mice. Table 1 shows that Akita mice had a higher blood pressure than the controls. As expected, administration of telmisartan significantly lowered the blood pressure. Compared to the controls, Akita mice also had considerably higher levels of blood glucose and HbA1c, which eventually led to loss of body weight. Telmisartan decreased the blood glucose level and led to an increase in body weight in Akita mice (Table 1). The urinary albumin excretions were significantly increased in untreated Akita mice compared to wild-type controls, and administration of telmisartan significantly reduced urinary albumin excretion (Table 1).

**Figure 1:**
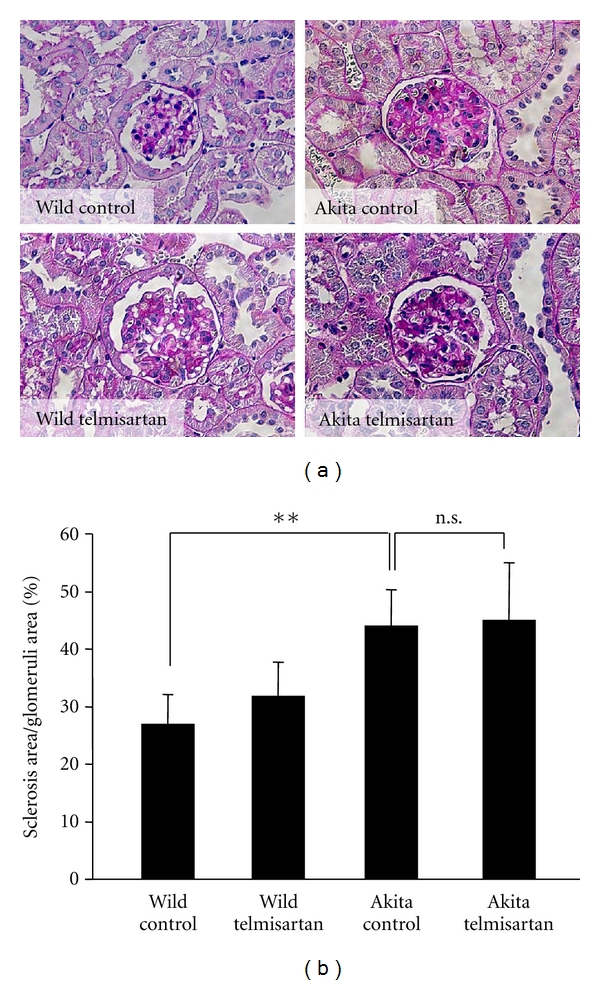
Morphometric analyses of the glomeruli of Akita mice. (a) Eight-week-old Akita mice and control mice received telmisartan (5 mg·kg^−1^·day^−1^, in their drinking water) or no treatment, respectively, for 15 weeks (*n* = 8 in each group). After 15 weeks, the mice were sacrificed, the kidneys were harvested, and periodic acid-Schiff staining was performed. (b) Quantification of sclerosis per glomerular area was performed with the ImageJ software. **P < 0.01, *n.s*.: not significant.

Next, we investigated the effect of telmisartan on the glomerular morphology. Expansion of the mesangial areas was observed in Akita mice; however, telmisartan had no profound effect on the glomerular morphology as determined by light microscopy (Figure 1).

### 3.2. Telmisartan Inhibits the Notch Pathway and the Expression of TGF-*β*, Which Are Activated in the Glomeruli of Akita Mice

**Figure 2:**
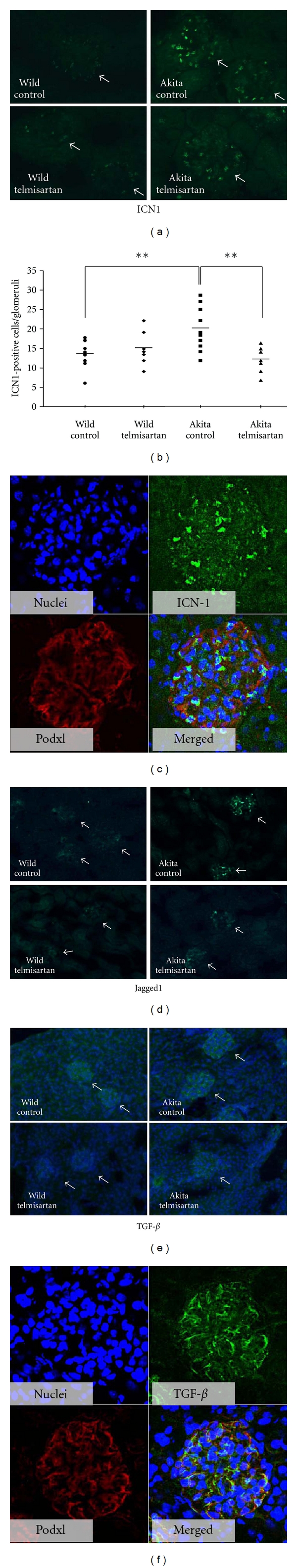
Notch pathway was activated in the glomeruli of Akita diabetic mice and telmisartan inhibited its expression. The expression of the intracellular domain of Notch1 (ICN1) (a and c), Jagged1 (d), and transforming growth factor *β* (TGF-*β*) (e and f) were examined by immunohistochemistry. Anti-podocalyxin (Podxl) antibody was used as a marker for podocyte. ICN-1 was localized to podocyte nuclei (c), while TGF-*β* was localized to podocyte cytoplasm, respectively (f). Quantification of ICN1-positive cells per glomeruli was performed (b). Ten glomeruli of each specimen were randomly selected. The ICN1-positive cells within the glomeruli were counted under a fluorescence microscope. Statistical significance was analyzed using Student's *t*-test. Arrows indicated the glomerulus. Bars indicated the mean value. **P < 0.01.

Recently, it has been reported that the Notch pathway is activated in podocytes in DM. Therefore, we examined the Notch pathway in Akita mice. ICN1 staining in kidneys revealed that the number of ICN1-positive cells in the glomeruli was significantly higher in Akita mice (Figures 2(a) and 2(b)). We could not observe ICN1-positive cells other than in the glomeruli. This indicated that the Notch pathway was activated in Akita mice, and the activation of the Notch pathway seemed to be restricted to the glomeruli. In order to identify cell types that were activated by the Notch pathway within the glomeruli, we also carried out coimmunostaining with an anti-ICN1 antibody and an anti-podocalyxin antibody (a marker for podocytes). We localized ICN1 proteins to the nuclei of the cells which were positive for podocalyxin within the cytoplasm (Figure 2(c)). Therefore, Notch pathway was activated in podocytes in diabetic conditions. Administration of telmisartan significantly reduced the number of ICN1-positive cells in the glomeruli (Figures 1(a) and 1(b)). Next, we investigated the expression of Jagged1, which is a ligand for the Notch receptor. The expression pattern of Jagged1 was quite similar to that of ICN1 (Figure 2(d)). These results indicated that telmisartan inhibited the Notch pathway *in vivo* either directly or indirectly. It has been reported that the Notch pathway in podocytes was activated by TGF-*β* signaling [8]. Therefore, we investigated the expression of TGF-*β* by immunohistochemistry. We observed upregulated TGF-*β* expression in the glomeruli of Akita mice (Figure 2(e)), especially in podocytes (Figure 2(f)). Administration of telmisartan also suppressed the expression of TGF-*β* in the glomeruli (Figure 2(e)).

### 3.3. Angiotensin II Activates the Notch Signaling Pathway through Increased Expression of TGF-*β* and VEGF-A in Cultured Podocytes

**Figure 3:**
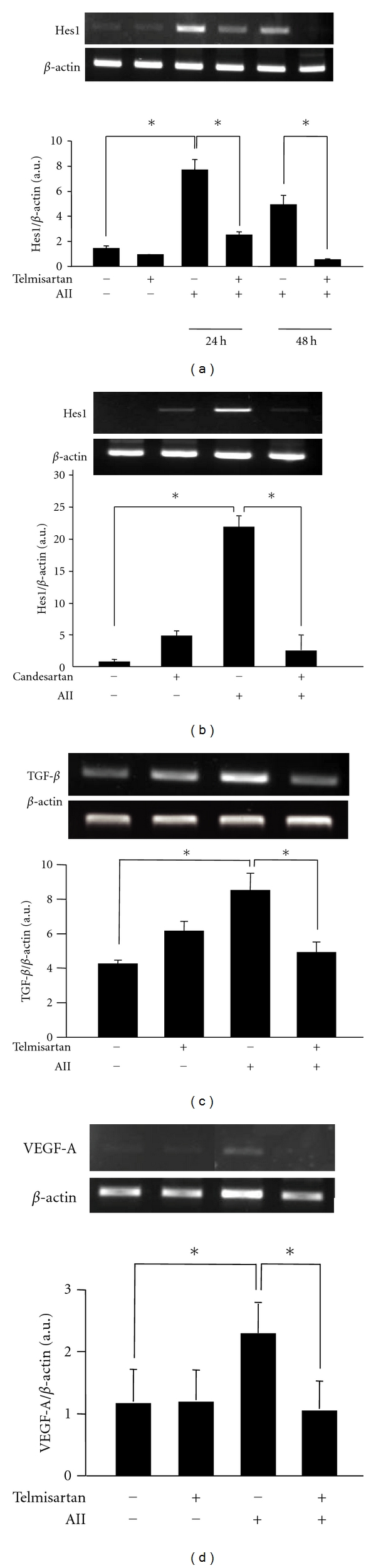
Telmisartan suppressed the activation of the Notch signaling pathway through inhibition of the angiotensin II type 1 receptor. The mRNA expression of Hes1, one of the Notch target genes; transforming growth factor *β* (TGF-*β*); vascular endothelial growth factor-A (VEGF-A) were examined by reverse transcriptase-polymerase chain reaction. (a) The podocytes were stimulated with 10^−6 ^M Angiotensin II (AII) for 24 to 48 h. The mRNA expression of Hes1 increased in the presence of AII and peaked at 24 h. On the other hand, 10^−6 ^M telmisartan suppressed the AII-induced mRNA expression of Hes1 (upper panel). Quantification of the Hes1 mRNA expression compared to the internal control (*β*-actin) (lower panel). (b) The podocytes were treated with 10^−6 ^M AII in the presence or absence of 10^−8 ^M candesartan for 24 h. Candesartan also suppressed the AII-induced mRNA expression of Hes1. (c) AII increased the TGF-*β* mRNA by 2.5-fold within 12 h. Telmisartan (10^−6 ^M) suppressed the expression of TGF-*β* significantly. (d) AII increased the VEGF-A expression by 2.0-fold. Telmisartan suppressed the expression of VEGF-A significantly. *P < 0.05.

Telmisartan lowered the blood pressure and improved the blood glucose level in Akita mice. From these findings, we were not able to completely exclude the possibility that the inhibitory effect of telmisartan on the Notch pathway* in vivo *was due to a systemic effect. Therefore, we used cultured mouse podocytes that were conditionally immortalized in order to not only rule out the influence of blood pressure and glucose levels but also elucidate the mechanism by which telmisartan inhibits the Notch pathway. Telmisartan is an AT1R blocker. For this reason, we studied the effect of angiotensin II (AII), a ligand for AT1R, on the activation of the Notch pathway. As shown in Figure 3(a), the mRNA expression of hairy enhancer of split homolog-1 (Hes1), which was a target gene of the Notch signaling pathway, increased considerably in the presence of 10^−6 ^M AII. In addition, telmisartan inhibited the AII-induced mRNA expression of Hes1 (Figure 3(a)). The expression of Jagged1 mRNA was also increased in the presence of AII, and telmisartan inhibited AII-induced mRNA expression of Jagged1 (data not shown). We also examined the effect of candesartan, another type of AT1R blocker, and found that candesartan inhibited the AII-induced mRNA expression of Hes1 same as telmisartan (Figure 3(b)). It has been reported that TGF-*β* and VEGF-A activate the Notch pathway [12]; therefore, the effect of AII on the expression of TGF-*β* and VEGF-A was investigated. As shown in Figures 3(c) and 3(d), incubation with AII significantly increased the expression of both TGF-*β* and VEGF-A. Telmisartan reversed this effect. 

**Figure 4:**
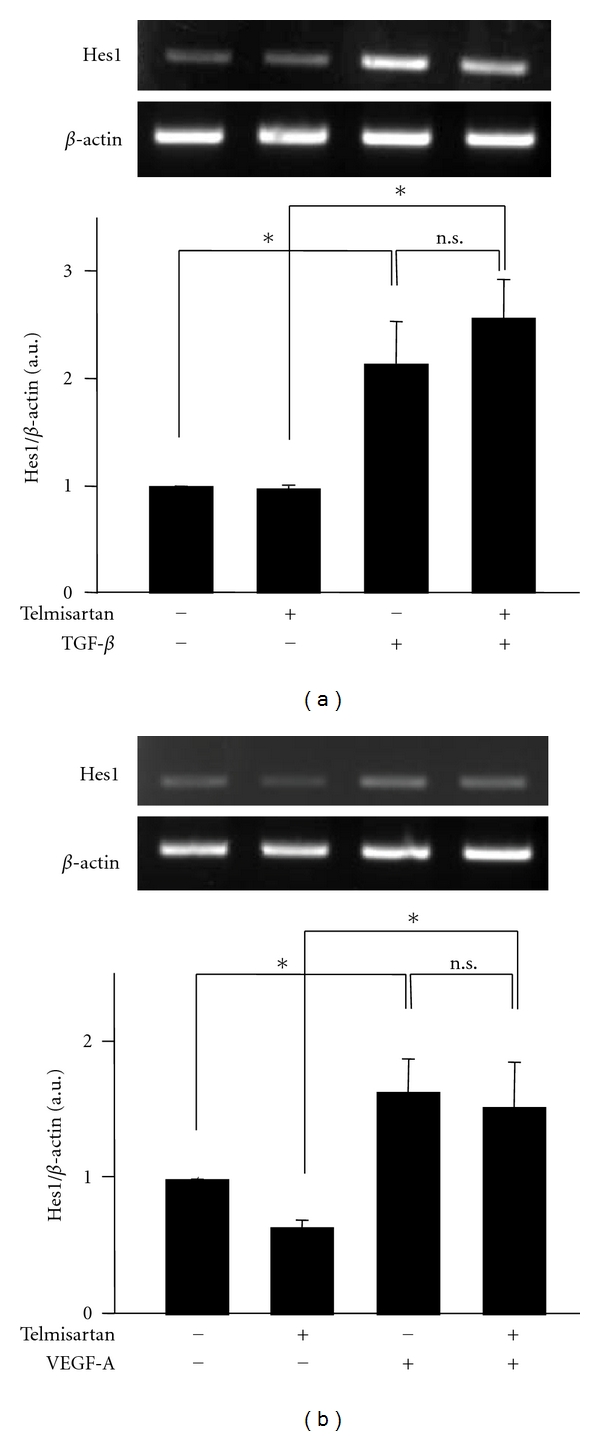
TGF-*β* and VEGF-A directly activated the Notch pathway. The podocytes were stimulated with 5 ng/mL transforming growth factor *β* (TGF-*β*) or 10 ng/mL vascular endothelial growth factor-A (VEGF-A) in the presence or absence of 10^−6 ^M telmisartan. The mRNA expression of Hes1 was examined by reverse transcriptase-polymerase chain reaction. (a) TGF-*β* increased the expression of Hes1 irrespective of the presence or absence of telmisartan (upper panel). Quantification of Hes1 expression compared to the internal control (*β*-actin). TGF-*β* significantly increased the Hes1 expression within 2 h by 2.1-fold (lower panel). (b) VEGF-A increased the expression of Hes1 irrespective of the presence or absence of telmisartan (upper panel). Quantification of the Hes1 expression compared to the internal control (*β*-actin). VEGF-A significantly increased the Hes1 expression within 2 h by 1.6-fold (lower panel). *P < 0.05, *n.s.*: not significant.

Finally, we observed the effects of TGF-*β* and VEGF-A on the activation of the Notch pathway and found that these growth factors could activate the Notch pathway. However, telmisartan had no effect on the Notch pathway in the presence of TGF-*β* or VEGF-A (Figure 4).

### 3.4. Telmisartan Suppresses the Podocyte Apoptosis Induced by Angiotensin II

**Figure 5:**
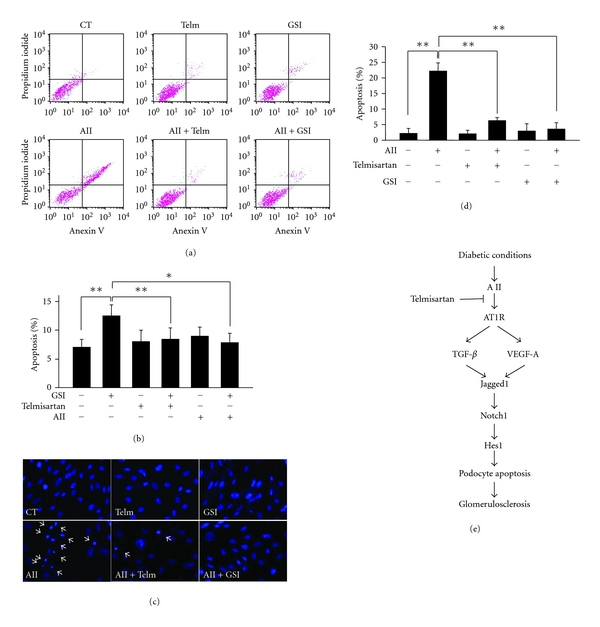
Telmisartan suppressed the podocyte apoptosis which was induced by angiotensin II. The effects of AII as well as telmisartan on the podocytes apoptosis were examined by the flow cytometry or by the Hoechst staining. (a, b) The podocytes were treated with 10^−6 ^M AII in the presence or absence of 10^−6 ^M telmisartan or 5 mM *γ*-secretase inhibitor (GSI) for 72 h. Apoptosis in podocytes was determined by low propidium iodide staining and prominent annexin V labeling using the flow cytometry. AII significantly induced podocytes apoptosis compared to the controls (12.56 ± 1.9% versus 7.09 ± 1.4%). Telmisartan significantly suppressed AII-induced apoptosis in podocytes (8.51 ± 2.0% versus 12.56 ± 1.9%). GSI also significantly suppressed that (7.89 ± 1.6% versus 12.56 ± 1.9%). Representative results of three independent experiments were presented. *P < 0.05, **P < 0.01. (c) The apoptosis in podocytes was examined by Hoechst staining. The podocytes were treated with 10^−6 ^M AII, 10^−6 ^M telmisartan, and 5 mM GSI as indicated in the figures for 72 h. Apoptosis was determined by nuclear condensation pattern and expressed as the percentage of apoptotic cells per high-power field. A total of 5 high-power fields in a pericentric distribution were quantitated per well. (d) Telmisartan and GSIs suppressed the podocyte apoptosis (CT 2.3 ± 1.5%, AII 22.3 ± 2.54%, Telm + AII 6.3 ± 0.9%, and GSI + AII 3.6 ± 2.0, resp.). *Telm*: telmisartan, **P < 0.01. (e) Schematic illustration of the effects of telmisartan on the Notch pathway in podocytes.

It has been reported that the activated Notch pathway induces apoptosis to the glomerular podocytes which eventually causes glomerulosclerosis. Therefore, we investigated whether telmisartan could prevent podocyte apoptosis. As shown in Figures 5(a) and 5(b), flow cytometer studies using annexin V and propidium iodide showed that apoptotic cells were increased in the podocytes treated with AII (12.56 ± 1.9% versus 7.09 ± 1.4% in the control group, *P* < 0.01), and telmisartan treatment significantly decreased the AII-induced apoptotic cells (8.51 ± 2.0% versus 12.56 ± 1.9% in the AII group, *P* < 0.01). We also examined the apoptosis by the use of Hoechst 33342 staining as shown in Figures 5(c) and 5(d). Nuclear condensation was observed in the podocytes in the presence of AII, and those changes were significantly decreased when the podocytes were treated with telmisartan. We also examined the effects of *γ*-secretase inhibitor (GSI) on the AII-induced apoptosis and found that GSI, an inhibitor of Notch signaling, was able to inhibit the AII-induced apoptosis (Figure 4). Collectively, these results indicated that the AII induced podocytes apoptosis via the activating Notch signaling pathway, and telmisartan inhibited podocytes apoptosis through the inhibition of Notch signaling pathway (Figure 5(e)).

## 4. Discussion

In the present study, we investigated the activation of the Notch pathway in the glomeruli (especially in the podocytes) of Akita mice. Treatment with telmisartan significantly reduced not only the urinary albumin excretion which was usually seen as an early manifestation of diabetic nephropathy but also the activation of the Notch pathway. We also confirmed that AII induced the activation of the Notch pathway in cultured podocytes. Incubation with AII increased the expression of TGF-*β* and VEGF-A, and telmisartan reversed this effect. TGF-*β* and VEGF-A could directly activate the Notch pathway.

Diabetic nephropathy, the leading cause of ESRD in the western world and Asia, is a considerable socioeconomic burden. Investigation of the pathophysiology and establishment of a treatment for diabetic nephropathy is urgently needed. AII is a potent vasoconstrictor hormone that is cleaved from angiotensinogen by renin and ACE. In addition to its known vital role in both cardiovascular and blood pressure homeostasis, several lines of evidence implicate a role in diabetic nephropathy. Durvasula and Shankland have reported that high glucose activates the local RAS in podocytes (independent of ACE activity), which led to injury of the podocytes [13]. Therefore, RAS are locally and systemically activated under diabetic conditions. It has also been reported that the injury of podocytes, referred to as podocytopathy, is a hallmark not only in diabetic nephropathy but also in virtually all glomerular diseases [14]. There are not many pharmacological options to treat diabetic nephropathy; ACEIs and/or ARBs are currently the only drugs that effectively slow the progression of diabetic nephropathy [15]. Furthermore, clinical trials demonstrated that ARBs also lower the risk of type 2 DM compared with other antihypertensive therapies. These observations indicate that ARBs can potentially be used to induce effects other than blood pressure lowering effects. Indeed, ARBs have recently been proven to attenuate inflammation and oxidative stress and inhibit apoptosis [16]. These effects are known as pleiotropic effects. In addition to the previously reported pleiotropic effects, in the present study, we identified that telmisartan inhibited the activation of the Notch pathway. The Notch pathway is known to control a number of cell-fate-specific events in multiple organisms, especially during development, and it also plays a crucial role in diseases such as cancers and autoimmune diseases [17]. It has been recently reported that the Notch pathway is activated in mouse models of DM such as Lpr^db/db^ mice (which mimics type 2 DM), in streptozotocin-treated mice (which leads to type 1 DM), and in kidney specimens from patients with DM [8]. It has also been reported that high glucose activated Notch pathway and increased the expression of VEGF in cultured podocyte [18]. We confirmed the activation of the Notch pathway in another diabetic animal, the Akita mouse. Our findings support the idea that the Notch pathway is generally activated in podocytes in DM. In recent years, GSIs received significant attention as drug candidates for the treatment of Alzheimer's disease and cancers [19]. Since GSIs are capable of inhibiting the Notch signaling pathway, they can be used in the treatment of diabetic nephropathy in the future. In addition to GSIs, our data also suggest that telmisartan inhibits the Notch pathway. To the best of our knowledge, this is the first report that describes the ARB-induced inhibition of the Notch pathway both *in vivo* and *in vitro*. Telmisartan is a potent and highly selective AT1R antagonist. Furthermore, telmisartan exerted effects other than the blockade of AT1R, such as PPAR*γ* activation [20]. Our data showed that telmisartan improved the levels of blood glucose, which might indicate that telmisartan functioned as a PPAR*γ* agonist and improved insulin resistance in Akita mice. Although telmisartan significantly reduced urinary albumin excretion, we were not able to detect profound histological improvement. There might be some time difference between the improvement in urinary albumin excretion and the improvement histologically. Telmisartan lowered the blood pressure and improved the blood glucose level in Akita mice. From these findings, we were not able to completely exclude the possibility that the inhibitory effect of telmisartan on the Notch pathway* in vivo *was due to a systemic effect. However, we also used cultured podocytes in order to rule out the influence of blood pressure and glucose levels. Therefore, we argue that telmisartan could directly affect podocytes in order to inhibit the Notch pathway. We also investigated whether candesartan, another ARB, could suppress the Notch pathway and found that candesartan also inhibited Notch signaling pathway. Therefore, the inhibitory effect of Notch pathway by telmisartan seems to be a class effect of ARB.

It has been reported that the genetically activated Notch pathway in podocytes in mice activated p53 and induced apoptosis, which led to decreased expression of the slit diaphragm-related protein such as nephrin, causing proteinuria and renal dysfunction [8]. We tried to detect apoptosis by terminal deoxyribonucleotidyl transferase dUTP nick-end labeling (TUNEL) staining and by staining for activated caspase 3. However, we could not observe apoptosis in the glomeruli of Akita mice, and this could be attributed to technical reasons.

There are some limitations to this present study. First, we were not able to completely exclude the possibility systemic effects of telmisartan for reducing Notch signal *in vivo*. Second, we are not able to explain the reason why telmisartan did not improve the glomerulosclerosis which was seen in Akita mice. Third, we still do not completely understand the biological significance of activated Notch pathway in diabetic condition.

In summary, we showed that the Notch pathway was activated in podocytes of Akita mice and that administration of telmisartan inhibited the Notch pathway. Our data might indicate that telmisartan inhibits the Notch pathway. In addition to its blood pressure lowering effect, which leads to reduced cardiovascular morbidity and mortality, telmisartan might improve the renal prognosis, especially in diabetic subpopulations. Further investigations are needed to prove this hypothesis in the future.

## Figures and Tables

**Table 1:** Characteristics of the experimental groups of mice.

	Wild control	Wild telmisartan	Akita control	Akita telmisartan
Blood glucose (mg/dL)	250 ± 34	284 ± 58	1216 ± 130*	955 ± 137^∗,†^
HbA1c (%)	4.3 ± 0.3	4.2 ± 0.3	10.8 ± 1.4*	11.8 ± 0.5*
Body weight (g)	36.4 ± 3.4	40.7 ± 9.0	20.8 ± 0.8*	23.2 ± 1.4^∗,†^
Systolic blood pressure (mmHg)	109.3 ± 4.7	96.1 ± 7.3	126.4 ± 5.9*	110 ± 5.1^∗,†^
Urinary albumin (mg/day)	21.2 ± 9.4	10.9 ± 2.51	51.4 ± 11.6*	33.8 ± 8.5^∗,†^

Data are expressed as the mean ± standard deviation (SD). *P < 0.01 versus wild-type control, ^†^ *P* < 0.01 versus Akita control.
